# Artificial Scaffold Polypeptides As an Efficient Tool for the Targeted Delivery of Nanostructures In Vitro and In Vivo

**DOI:** 10.32607/actanaturae.11545

**Published:** 2022

**Authors:** V. O. Shipunova, S. M. Deyev

**Affiliations:** Shemyakin-Ovchinnikov Institute of Bioorganic Chemistry of the Russian Academy of Sciences, Moscow, 117997 Russia

**Keywords:** nanoparticles, DARPins, affibody, anticalins, scaffold proteins, ADAPT, HER2, HER1, EGFR, EpCAM, conjugation, targeted delivery

## Abstract

The use of traditional tools for the targeted delivery of nanostructures, such as antibodies, transferrin, lectins, or aptamers, often leads to an entire range of undesirable effects. The large size of antibodies often does not allow one to reach the required number of molecules on the surface of nanostructures during modification, and the constant domains of heavy chains, due to their effector functions, can induce phagocytosis. In the recent two decades, targeted polypeptide scaffold molecules of a non-immunoglobulin nature, antibody mimetics, have emerged as much more effective targeting tools. They are small in size (3–20 kDa), possess high affinity (from subnano- to femtomolar binding constants), low immunogenicity, and exceptional thermodynamic stability. These molecules can be effectively produced in bacterial cells, and, using genetic engineering manipulations, it is possible to create multispecific fusion proteins for the targeting of nanoparticles to cells with a given molecular portrait, which makes scaffold polypeptides an optimal tool for theranostics.

## 1. INTRODUCTION

Developing novel, highly sensitive diagnostic tools and targeted cancer therapies, as well as improving on the existing ones, is among the main drivers of developments in modern nanobiomedicine. Targeted drug delivery is the key issue in theranostics, with respect to the novel approaches to the design of drugs that would simultaneously act as early diagnostic tools, therapeutic agents, and tools for the monitoring of treatment effectiveness [1, 2].

**Fig. 1:**
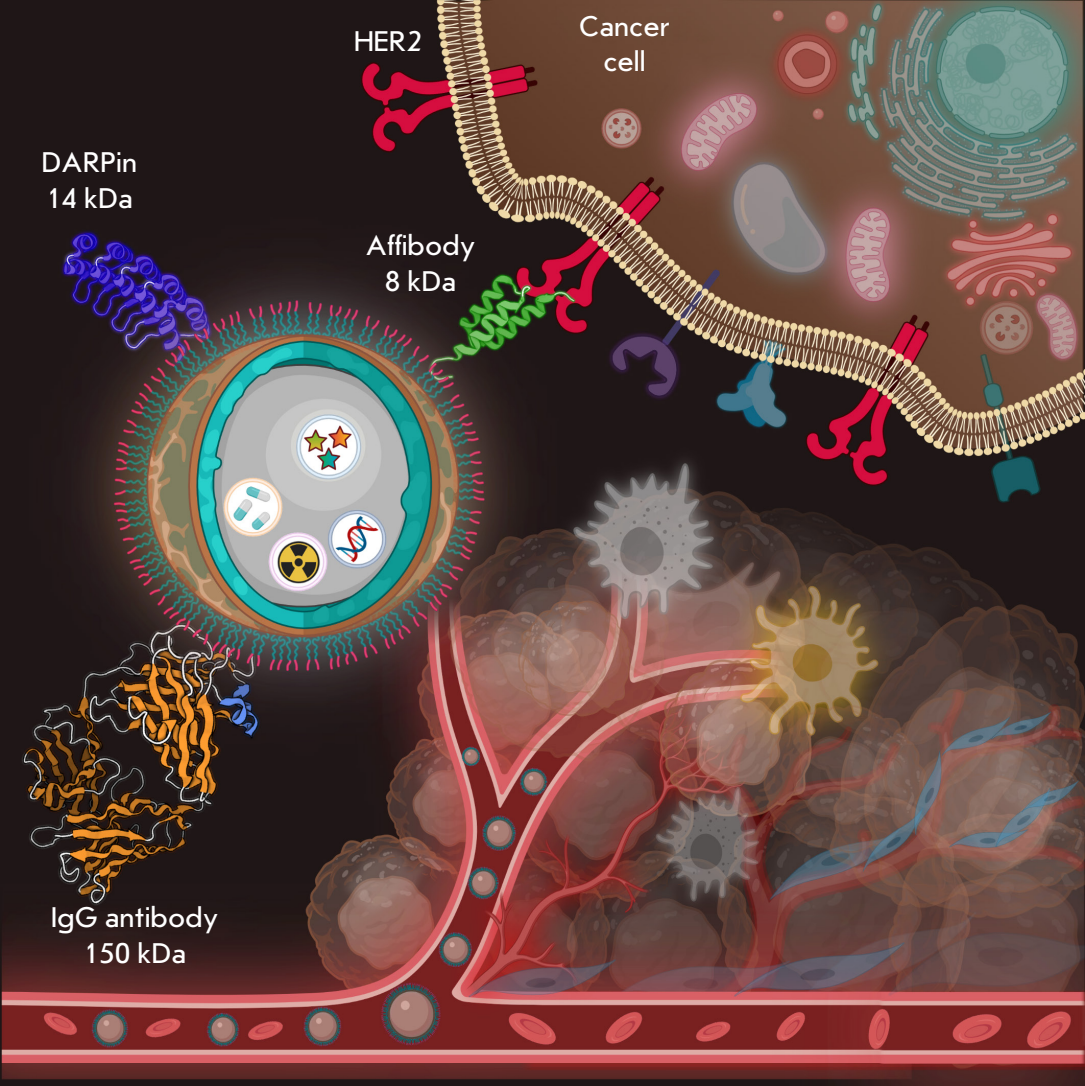
Nanoparticles as a platform for the design of theranostics tools. The scheme shows a core–shell nanoparticle, which is a matrix for loading both diagnostic (fluorescent or radioactive) and therapeutic compounds (chemotherapeutic substances and genes). The nanoparticle surface is modified with various targeting compounds: antibodies (IgG, 150 kDa) or scaffold polypeptides (DARPins (14 kDa) or affibodies (8 kDa)) are conventionally used. The diagram shows the nanostructures targeting the HER2 tumor marker, which is overexpressed on the surface of human breast cancer cells. The plot was created using Biorender.com

Nanoparticles differing in their nature are promising objects for the design of theranostic agents (Fig. 1). Nanoparticles possess a broad range of unique characteristics: they are small in size, boast a high ratio of surface area to the number of bulky atoms and can form nanoparticle–ligand complexes, including those with compounds larger than their own size (such as proteins, various drugs, etc.) and selectively deliver them to a specific target, thus implementing the targeted delivery strategy. These, and many other, advantages make nanoparticles excellent diagnostic and therapeutic agents in various areas of medicine (in particular, for the detection and optical imaging of malignant tumors and targeted drug delivery). However, several factors limit the successful implementation of nanobiocomplexes in clinical practice. In particular, constructs that are characterized by minimal toxicity, high specificity in target recognition, and maximum therapeutic and targeting efficacy are not always available. Meanwhile, such complexes are expected to be characterized by low immunogenicity so as to make possible the performing of multiple courses of therapy.

More than 20 nanoparticle-based drugs are currently used for tumor treatment, and a number of agents are in the late phases of clinical trials. The efficacy of these drugs (e.g., liposomal doxorubicin Myocet (non-PEGylated liposomal formulation) or Caelix (polyethylene glycol-coated liposomal doxorubicin)) or micellar paclitaxel (Genexol-PM) is based on the effect of enhanced permeability and retention (EPR) of tumor vessels. Because there is high demand for oxygen and blood supply, a new vascular network develops in the tumor. This network is constituted by defective endothelial cells with wide fenestrations (up to 4 µm); the vessels do not possess a smooth muscle layer, and endothelial cells lack angiotensin II receptors. Due to the impaired lymphatic efflux observed in the 150- to 200-µm tumor cell aggregates surrounded by this vascular network, molecules and nanostructures with a size of up to 150 nm can stay near the tumor and exert their therapeutic effect.

However, the EPR effect is characterized by significant heterogeneity (both between different tumor models and even within the same tumor) and is pronounced much stronger in rodents with tumor xenografts than it is in human tumors. This is related to the slower tumor growth rate in humans and formation of a normal vascular network with a well-developed lymphatic efflux compared to rapidly proliferating tumors in rodents [3, 4]. Meanwhile, even for a really strong EPR effect (e.g., for rapidly progressive Kaposi sarcoma), only a small number of injected nanoparticles ( < 0.7%) get inside a tumor [5]. The following nanobiomedicine-related problems are yet to be solved: the treatment of aggressive metastatic cancer [6], integration of the methods of personalized noninvasive diagnosis and therapy [7], and the generation of physiologically relevant xenograft animal models [4].

There exist different approaches to targeted drug delivery to the tumor, which mainly consist in improving the efficiency in their binding to cancer cells, endothelial cells or immune cells [8], as well as drug internalization by the cell and its controlled release (including upon exposure to external factors: light, pH, temperature, electromagnetic fields, etc.) [9, 10, 11, 12, 13]. The nanostructure surface is modified using targeting agents of differing nature, such as antibodies and their derivatives [14, 15], transferrin, the epidermal growth factor, lectins [16], molecules based on DNA/RNA (aptamers and protein–nucleic acids), low-molecular-weight compounds (folic acid, saccharides (e.g., galactosamine)), etc.

The application of these molecules elicits a full range of undesirable effects. Thus, the large size of IgG antibodies often prevents an efficient use of the surface of modified nanostructures; the heavy chain constant domains exhibit effector functions that can induce phagocytosis and cause inflammation without being involved in selective target recognition, or induce undesirable in vivo immunomodulation. The size of an antibody limits the diffusion of its molecules deep inside a tumor.

Targeted polypeptide scaffold molecules of a non-immunoglobulin nature, which are produced by phage, cell surface, or ribosome display techniques, appear to be more efficient tools in targeting nanostructures to target cells. These polypeptides are produced by mutagenesis of the protein motifs involved in the protein–protein interactions in living systems. Affibodies and DARPins are the most vivid examples of this group of targeting compounds (Fig. 1).

## 2. THE MAIN STRUCTURAL CHARACTERISTICS OF SCAFFOLD PROTEINS AND THEIR ADVANTAGES OVER FULL-LENGTH ANTIBODIES

The hybrid technology for producing monoclonal antibodies, which was described by Georges Köhler and César Milstein and for which they were awarded the Nobel Prize in Physiology or Medicine in 1984, has enabled significant advances in the implementation of the concept of the "magic bullet." This concept was formulated by Paul Ehrlich and consists in developing an efficient way to deliver a therapeutic agent exclusively to the disease site without affecting healthy tissues or triggering undesired harmful effects. More than eight dozen antibodies have been clinically tested and approved for clinical use. However, even these antibodies cause a broad range of undesirable effects, which has inspired intensive efforts in synthetic biology focusing on the design of recognition scaffold proteins.

Various recognition scaffold proteins have been designed over the past 20 years, largely thanks to the synthetic library technology. Similar to antibodies, these proteins have a conserved scaffold region and a variable recognition region. Specifically binding scaffold proteins are usually designed using combinatorial libraries that contain sets of genes differing in their variable regions. In particular, proteins based on the domains of lipocalin, zinc fingers, Src homology domains, PDZ domains, Kunitz-type serine protease inhibitor domains, cystatins, DNA-binding protein Sac7d, A-domains of various membrane receptors, gamma-B-crystallin and ubiquitin-binding domains, etc. are being developed. More than 20 classes of antibody mimetics have been designed thus far; the key ones are listed in Table.

**Table:** The key representatives of scaffold proteins (antibody mimetics)

Proteins	Protein platform: a scaffold	Molecular weight, kDa	Representative eferences
Avimers	Domain A of extracellular receptors	4	[17, 18]
Adhirons	Phytocistatin domain	10	[19]
Adnectins (monobodies)	Fibronectin type III domain (FN3)	10	[20, 21, 22]
Atrimers	Tetranectin CTLD	60–70	[23]
Anticalins	Lipocalin domains	20	[24, 25]
Affibodies	Z domain of protein A derived from the Staphylococcus aureus cell wall	6	[26]
Affilins	Gamma-B-crystallin/ubiquitin domains	20/10	[27, 28]
Affimers	Domains of cystatin, a cysteine protease inhibitor	12	[29, 30]
Affitins (Nanofitins)	Domains of DNA-binding protein Sac7d	7	[31, 32, 33]
DARPins	Drosophila ankyrin repeat	14–18	[34, 35, 36, 37]
Knottins	Disulfide-rich peptide toxins	3	[38]
OBodies	Aspartyl-tRNA synthetase anticodon recognition domain	10	[39]
Kunitz domain polypeptides	Kunitz domain of serine proteases	6	[40]
Pronectins	Human fibronectin domain 14	75	[41]
Repebodies	Leucin-rich repeats of variable lymphocyte receptors	3–30	[42]
Fynomers	SH3-domain of Fyn kinase (Src homology domain)	7	[43]
Centyrins	FN3 domains of tenascin C	9	[44]
ADAPT (ABD-Derived Affinity Proteins)	Albumin-binding domain of protein G	5	[45]
NanoCLAMP (nano-CLostridial Antibody Mimetic Proteins)	Carbohydrate-binding domain of hyaluronidase of Clostridium perfringens hyaluronidase	16	[46]
ARM (Armadillo Repeat Proteins)	Drosophila proteins carrying the armadillo domain	39	[47]
PDZ proteins	PSD-95/Discs-large/ZO-1 domains	10	[48]

The aforementioned proteins are small in size (8–20 kDa) and are characterized by high affinity for molecular targets (subnano–femtomolar binding constants), as well as optimal biochemical and thermodynamic characteristics. They remain stable for a long time at high temperatures (up to 80°C), low pH (up to pH 2), and upon exposure to chaotropic agents. The incorporation of cysteine residues into these proteins both yields dimers with target characteristics and allows one to perform regioselective protein modification using various compounds via disulfide bond formation. The low immunogenicity of proteins due to their synthetic nature allows one to use them for therapeutic purposes, especially when a single therapy course is insufficient for achieving remission and repeated injection of the drug is needed.

**Fig. 2:**
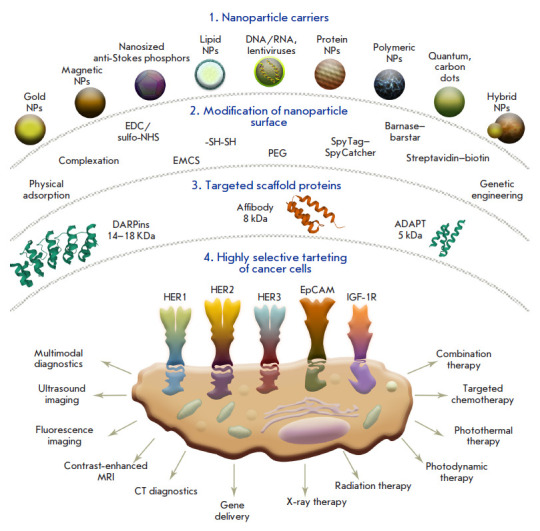
Artificial scaffold polypeptides for the targeted delivery of nanocarriers to target cells. **1 **– A wide range of nanoparticles that are used for diagnostic and therapeutic applications. **2 **– Methods for surface modification with targeting molecules: physical adsorption, chemical conjugation, protein adapter systems, and genetic engineering. **3 **– Scaffold proteins already used for the targeted delivery of nanoparticles: DARPins, affibodies, and ADAPT. **4 **– Targeted delivery of nanoparticles to the receptors of cancer cells for different applications: diagnostics, including the multimodal one, and therapy, including the combined one

All classes of these proteins have free N- and C-termini lying outside the recognition sequence, which enables efficient chemical conjugation of the proteins to the polymers on the nanoparticle surface, as well as the production of genetically engineered constructs (such as fusion proteins consisting of scaffolds and protein toxins) for therapeutic applications. The small size of scaffolds makes it possible to significantly increase the number of their molecules tethered to the nanoparticle surface compared to IgG. Only DARPins, affibodies and albumin-binding domain (ABD) derivatives are commonly used today for the delivery of nanoparticles to molecular targets (Fig. 2). A number of studies focusing on the engineering of nanoparticles for targeted delivery based on repebodies [49, 50, 51], affimers [52], affitins [53, 54, 55], and knottins [56] have also been conducted.

## 3. DARPINS AS A TOOL FOR THE TARGETED DELIVERY OF NANOPARTICLES

DARPins (Designed Ankyrin Repeat Proteins), or ankyrin repeat proteins, are unique tools for solving problems related to personified medicine and fundamental research in molecular and cellular biology [57, 58]. These proteins are based on ankyrin repeats: a series of tightly packed repeats, each consisting of approximately 33 amino acid residues. In turn, each repeat consists of two α-helices connected by a short loop and one β-turn joining this repeat to the next one. Proteins with ankyrin repeats form a dexiotropic solenoid that contains a long hydrophobic backbone and a hydrophilic surface accessible to the solvent [59]. They often mediate protein– protein interactions inside the cell (e.g., when acting as cytoskeleton proteins, transcriptional initiators or cell cycle regulators). Proteins carrying four to six repeats commonly occur in nature, but sometimes the number of repeats can exceed 29. Seven amino acid residues in the repeat (six residues in the β-turn and one in the helix) form the binding surface. When constructing recombinant libraries, random substitutions are inserted into the codons encoding these residues. DARPins are often selected using the ribosome display technology. DARPins are typically formed by two or three (sometimes four) repeats sequentially located between the N- and C-termini. The molecular weight of these scaffold proteins depends on the number of repeats and is 14–18 kDa if a scaffold protein consists of two or three repeats. DARPins are extremely thermostable proteins that can withstand quite harsh conditions: heating to 90°C and exposure to proteases or chaotropic agents. DARPins specific for membrane-bound tumor markers (EpCAM, VEGF-A, HER2, as well as for the maltose-binding protein, MAP kinase, caspase 2, IgE antibody, and CD4) have been obtained [35, 60, 61, 62].

Since DARPins have a rather rigid framework and recognizing surface, steric challenges often occur upon target recognition. A novel, similar class of compounds, LoopDARPins, with soft protruding recognizing loops that do not disrupt the structure of the scaffold protein, has been designed to solve this problem [63].

**3.1. DARPins conjugated to magnetic nanostructures for targeted drug delivery**

A series of studies [64, 65, 66, 67, 68] have demonstrated that magnetic nanostructures represented by superparamagnetic iron oxide nanoparticles can be successfully modified with the DARPin G3 and DARPin 9_29 molecules [69], which selectively recognize the clinically relevant tumor marker HER2 (human epidermal growth factor receptor 2). DARPin_G3 binds to the domain IV of the HER2 receptor (residing in close proximity to the membrane) with K = 0.070 nM [61], while DARPin_9.29 binds to domain I of the HER2 receptor (being most distant from the membrane) with K = 3.8 nM [34].

The ERBB2 gene encoding HER2 plays an important role in the development of malignant tumors in humans. This gene is amplified in approximately 20–30% of all breast cancer cases and in many other types of malignant tumors. HER2 overexpression often correlates with a high metastatic potential and chemotherapy resistance of the tumor; it also presages a high risk of disease recurrence and a reduced overall survival rate for patients.

The HER2 molecule is already used as a target for the targeted therapy of breast and gastric cancer with humanized anti-HER2 antibodies: trastuzumab (Herceptin, Herclon) and pertuzumab (Perjeta, Omnitarg) [70, 71]. Unfortunately, the mechanisms related to the recruitment of complement molecules and cytotoxic leukocytes to cancer cells are insufficient to completely eliminate the tumor: so, targeted agents containing additional toxic compounds are needed. Thus, trastuzumab conjugated to a microtubule assembly inhibitor (trastuzumab emtansine, Kadcyla), which actually increases the effectiveness of the therapy for HER2-positive tumors, is used to treat HER2- positive breast and gastric cancer.

There is an urgent need for novel therapies for this disease that would be more effective. HER2-targeted nanoparticles exhibiting diagnostic and therapeutic properties seem to be among the most promising tools in our efforts to develop novel cancer treatment strategies.

Magnetic nanoparticles conjugated to DARPin G3 and DARPin 9_29 have proved to be effective for the theranostics of HER2-positive tumors. Thus, magnetic nanoparticles–DARPin G3 complexes targeting HER2 on the surface of the SK-BR-3 human breast adenocarcinoma cell line were obtained via chemical conjugation. This has enabled in vivo fluorescence and magnetic resonance imaging of HER2-overexpressing tumors [64].

Chemical conjugation of DARPin 9_29 to magnetic particles did not result in selective binding of nanoparticles to the target cells [67, 68]. Direct conjugation of small molecules to the nanoparticle surface seems to cause such problems as (1) partial protein denaturation on the nanoparticle surface, (2) binding through multiple amino groups and non-oriented attachment, and (3) steric hindrance upon target recognition. These problems have been solved using protein adapter systems. In particular, the high-affinity barnase:barstar protein pair was used as a mediator between the nanoparticle surface and the DARPin molecule.

The barnase-barstar pair is a unique tool for the design of multifunctional biomedical agents [72, 73, 74]. Barstar (10 kDa) is a natural inhibitor of barnase, a bacterial ribonuclease (12 kDa). These proteins are characterized by an extremely high affinity (the binding constant Kb ~ 1014 M –1) and fast interaction kinetics (the complexation rate constant kon ~ 108 M –1s–1), while the absence of these proteins in mammalian cells allows one to use them in the bloodstream without any potential complications related to competitive binding to endogenous blood components. Their small size and high binding constant make these proteins the ideal "molecular glue" in designing various self-assembling structures based on different modules, where one module (e.g., the therapeutic one) contains one component of the system (e.g., barstar), while the other module (e.g., DARPin) contains the other component (e.g., barnase). This "LEGO" approach allows one to avoid the standard problems related to chemical conjugation of the components to the nanostructures and obtain biologically active structures simply by mixing the components (e.g., nanoparticle–barstar + barnase–DARPin).

In particular, a novel, universal method for the biomodification of nanostructures of different nature has been developed; this method consists in using peptides that bind the solid phase and the barnase:barstar protein module [68]. It involves non-covalent modification of the nanoparticle surface with a peptide binding the SiO_2_ surface of nanoparticles (VKTQATSREEPPRLPSKHRPG)4VKTQTAS (silica-binding peptide, SBP) genetically fused to barstar (SBP-Bs). The efficiency of the proposed method was confirmed by the obtaining fluorescent and magnetic nanoparticles modified with DARPin 9_29 recognizing the HER2 tumor marker and by targeted delivery of these nanoparticles to the HER2-overexpressing cancer cells. Fusion proteins consisting of the SiO_2_-binding polypeptide and barstar (SBP-Bs), as well as those formed by DARPin 9_29 and barnase (DARPin 9_29-Bn), were produced and characterized to implement this approach. In both proteins, the functional modules are connected by a protease-resistant flexible peptide linker to preserve their functional activity. The targeted nanoparticles were obtained by self-assembly of two components: nanoparticles with barstar and the targeted DARPin with barnase. This approach turned out to be much more efficient in the recognition of the target (HER2 on the cell surface) compared to chemical conjugation.

This approach is rather versatile: the components of the adapter system containing barnase or barstar can be easily replaced. Twelve methods for the targeted delivery of nanoparticles modified with targeted polypeptides through barnase:barstar in different ways have been described [68]. The C-terminal motif of the Mms6 protein, one of the magnetosome membrane proteins in magnetotactic bacteria, was also used as a polypeptide that mediates protein binding in the barnase:barstar adapter system to the nanoparticle surface [67]. The self-assembled constructs based on MNPs-Bs-C-Mms6-DARP 9_29-Bn magnetite nanoparticles were used for targeted delivery to HER2-overexpressing SK-BR-3 cells. These constructs were shown to be efficient for selective in vitro labeling and imaging of HER2-positive cells [65, 67].

**3.2. Modification of gold nanostructures with DARPins**

Nanosized objects acquire unusual quantum chemical properties differing from those of large samples, making it possible to design multifunctional therapeutic and diagnostic tools [75, 76, 77, 78]. One of such interesting properties is the effect of localized surface plasmon resonance (LSPR) in gold and silver nanostructures, as well as in hybrid ones (e.g., core–shell nanostructures).

The LSPR phenomenon relies on the resonant excitation of plasmons (quasiparticles being quanta of free-electron vibrations at the interface between two phases having different refractive indices provided that the total internal reflection condition is met). If the conditions of LSPR are met, the intensity of the reflected light drops abruptly as the energy of the incident electromagnetic wave is transformed into plasmon energy. The absorbed energy can be converted into thermal energy: so, the hyperthermal properties of the sample with LSPR are implemented, which can be used in the therapy of tumors whose cells are highly sensitive to heating.

Basic research addresses the properties of LSPR nanostructures (mainly formed by gold and silver, as well as other, less conventional materials, such as aluminum, copper, palladium, and platinum). In particular, gold nanoparticles sized 5 nm and modified with DARP 9_29 have been obtained [79]. The non-covalent stabilization of uncoated gold nanoparticles using DARP 9_29 molecules has given rise to colloidally stable complexes containing target molecules capable of selective recognition of the surface of HER2-expressing cancer cells. A similar modification method has been used to produce gold nanorods 50 nm long and 7 nm in diameter for in vitro targeted delivery to HER2-positive cells and their selective destruction by photothermically induced local hyperthermia upon 20-min excitation (wavelength, 850 nm; intensity, 30 mW/cm2) [80]. The efficiency of the designed targeted nanorods for local hyperthermia has been confirmed by the fact that irradiation caused almost 100% death of exclusively HER2-overexpressing cells, while non-irradiated cells and cells exhibiting negligible HER2 expression remained fully viable.

**3.3. Modification of upconversion nanoparticles with DARPins**

Upconversion nanoparticles (nanosized anti-Stokes phosphors) are photoluminescent nanoparticles that convert lower-energy electromagnetic radiation (having a longer wavelength) into higher energy electromagnetic radiation (having a shorter wavelength) [81, 82, 83, 84, 85]. Nanosized anti-Stokes phosphors are NaYF_4_ crystals doped with rare-earth elements: namely, Yb^3+^, Er^3+^, and Tm^3+^. These nanostructures absorb several low-energy photons and re-emit one high-energy photon, thus implementing the upconversion phenomenon, where the emission wavelength is shifted toward shorter wavelengths (the blue shift or anti-Stokes shift) while most fluorescence processes in living systems involve the Stokes shift (the red shift). Nanosized phosphors are synthesized in such a way that excitation occurs in the biotissue transparency window (~ 980 nm), while emission occurs in the short-wave range suitable for most imaging devices to work with living objects both in vitro and in vivo. Nanosized anti-Stokes phosphors are excellent labels for in vivo imaging, since their long-lasting photoluminescence and time-resolved signal acquisition make it possible to completely eliminate biotissue autofluorescence and record a real signal without noise with a high sensitivity, so that even a single particle can be registered.

The NaYF_4_:Yb,Er,Tm/NaYF_4_ core/shell nanosized phosphors were coated with anti-HER2 DARPin DARPin 9_29 and used for targeted delivery to a HER2-positive cancer cell culture and for the imaging of tumor xenografts in animals for at least 24 h. A comprehensive preclinical study of the overall and specific toxicity of these nanostructures was performed, including an assessment of their allergenic, immunotoxic, and reprotoxic properties. The experimental results suggest that both non-targeted and targeted nanosized phosphors are functional, non-cytotoxic, biocompatible and safe for in vitro imaging of cells and in vivo imaging of tumors [86, 87, 88].

In order to ensure an additional therapeutic modality of nanophosphors, their surface was modified with the DARPin 9_29 protein fused with a low-immunogenicity fragment of the pseudomonad exotoxin A, LoPE, using genetic engineering techniques [89]. The resulting DARP-LoPE protein possesses all the qualities needed for theranostics: it is capable of targeted interaction with target cells and is cytotoxic upon binding to cells.

Exotoxin A of Pseudomonas aeruginosa (PE, ETA) is one of the most efficient apoptosis inducers owing to its enzymatic activity, which inhibits translation. PE consists of three domains: domain I is responsible for toxin binding and penetration into the cell; domain II participates in the intracellular transport of the toxin; and domain III possesses intrinsic enzymatic activity. It catalyzes the ADP-ribosylation of eukaryotic eEF2, thus inhibiting protein biosynthesis in the cell and eventually causing its death [90]. The truncated variants of this toxin (namely, the catalytic domain coupled to targeting modules characterized by different specificities) are used for designing efficient PE-based immunotoxins. HER2-recognizing DARPin-based immunotoxins coupled to a variant of the C-terminal (effector) fragment of PE (LoPE), with mutations in immunodominant human B-cell epitopes, have been obtained [91]. The immunogenicity and systemic toxicity of this fragment are lower than those of the unmodified fragment.

DARP-LoPE immunotoxin, a targeting cytotoxic protein, was conjugated to the surface of nanosized anti-Stokes phosphors using carbodiimide and an intermediate linker, polyethylene glycol. The as-synthesized nanosized phosphors were coated with PMAO, an alternating maleic anhydride–1-octadecene copolymer, and polyethylene glycol to ensure a greater colloidal stability [89]. The cytotoxicity of the targeting nanosized phosphor–PEG–DARP–LoPE complexes was studied for SK-BR-3-Kat cells. The half-maximum inhibitory concentration (IC_50_) of these complexes is 0.4 µg/mL, while IC_50_ = 200 µg/mL in the control CHO cells not expressing HER2, which proves that the resulting compounds exhibit targeted cytotoxicity.

Targeted cytotoxicity was significantly enhanced by using yttrium-90 as a beta emitter in nanosized phosphors. Radioactive nanosized anti-Stokes phosphors with a beta emitter (having a half-life of 63 h, which is optimal for biomedicine applications) and those modified with a DARP 9_29 fusion protein carrying a fragment of pseudomonad exotoxin A (PE) were synthesized [92]. Combining the two therapeutic modalities in a single nanoparticle yields a strong synergistic effect: the IC_50_ values of the targeted nanoparticles and nanoparticles doped with yttrium-90 were 5.2 and 140 µg/mL, respectively; the half-maximal inhibitory concentration of the nanoparticles containing a targeting module and yttrium-90 decreased significantly: IC_50_ = 0.0024 µg/mL [92].

**3.4. Lipid nanostructures conjugated to DARPins**

Lipid structures such as single-layered liposomes and exosomes were used as study objects to solve the problems of cancer theranostics.

Liposomes (117 ± 41 nm in size) loaded with an RNase barnase and chemically conjugated to anti-HER2 DARPin 9_29 were obtained [93, 94]. There is interest in RNases as a non-mutagenic alternative to the conventional chemotherapeutics. However, many mammalian RNases are not potent toxins, since they are significantly suppressed by the ribonuclease inhibitor that is present in the cytoplasm of mammalian cells. The ribonuclease barnase stands out, because it is not mutagenic, does not have severe toxic side effects, and once it has penetrated the cell, it cleaves RNA and causes cell death. The human ribonuclease inhibitor does not suppress barnase activity. Barnase causes degradation of low-molecular-weight RNAs (namely, tRNA and 5.8S rRNA, but not 5S rRNA). Upon internalization, barnase causes plasma membrane blebbing and apoptosis via internucleosomal chromatin cleavage. Therapy of HER2-positive BT474 xenograft tumors using liposomes loaded with barnase and modified with anti-HER2 DARPin in laboratory animals proved effective. The IC_50_ of barnase within the targeted liposomes was 0.11 nM for a BT474 cell culture in vitro; the in vivo efficacy of tumor growth inhibition was 84%. A combined treatment with the targeted liposomes and the targeted immunotoxin based on LoPE and DARPin EC1 inhibited tumor growth by 91.8% and completely prevented the appearance of metastases [94].

DARP EC1 binds to the EpCAM receptor with a picomolar affinity (Kd = 68 pM). EpCAM, a transmembrane glycoprotein with a molecular weight of 40 kDa and consisting of 314 amino acid residues, was first discovered as a tumor antigen in colon cancer cells in 1979. The key function of EpCAM is to provide intercellular communication. The EpCAM molecule is also often expressed in human breast cancer cells, which is associated with a poor prognosis. Thus, the findings of an extensive study showed that EpCAM expression is detected in 48% of human breast cancer cases [95]. Similarly to HER2, EpCAM is already employed as a target in monoclonal antibody-based immunotherapy (using Removab). In connection to this, it seems promising to combine different methods of affecting malignant tumors using scaffold proteins that target both HER2 and EpCAM to develop effective cancer treatment strategies.

Along with barnase-loaded liposomes, 90-nm liposomes loaded with PE40 and modified with DARP 9_29 were obtained [96]. These liposomes were used to selectively kill HER2-overexpressing cells (IC_50_ = 0.17 nM for SK-BR-3 cells and 0.21 nM for SK-OV-3 cells) [96].

An elegant approach to designing targeted lipid nanoparticles is to employ natural mechanisms for obtaining nanoparticles that have already been modified and do not require chemical conjugation. In particular, exosomes from HEK293T cells transduced with lentivirus, with the gene encoding HER2- detecting DARPin DARP G3 inserted, have been obtained [97]. These exosomes bound specifically to SK-BR-3 cells and have ensured targeted delivery of small interfering RNAs against the TPD52 gene, thus down-regulating the gene’s expression by 70% [97].

**3.5. Nucleic acid delivery using DARPins**

It has been demonstrated that DARPins can be used for the targeted delivery of genetic material into eukaryotic cells. Lentiviruses displaying HER2-targeting DARPins DARP G3, DARP H14R, DARP 9_29, DARP 9_26, DARP 9_16, and DARP 9_01 on their surface have been obtained [98]. DARPin 9_29 turned out to be the most effective DARPin both in terms of its content on the virus surface and the transduction of HER2-positive cells. DARPins were more effective than the previously used scFv mini-antibody, a HER2- targeting single-chain fragment of the light and heavy chains of 4D5 immunoglobulin [98].

DARPins were used to deliver small interfering RNAs complementary to mRNA of the Bcl-2 apoptosis regulator [99]. Dimers of DARPin EC1 fused with protamine 1, a small protein that forms a complex with nucleic acids, were used. Protamine 1 bound four to five small interfering RNA molecules and retained its specificity of binding to the EpCAM receptor on the MCF-7 cell surface. This made it possible to perform targeted transfection of exclusively EpCAM-overexpressing cancer cells and effectively inhibit the biosynthesis of Bcl-2 [98].

## 4. AFFIBODIES AS A TOOL FOR TARGETED NANOPARTICLE DELIVERY

Affibodies contain the Z domain of Staphylococcus aureus protein A, which consists of 58 amino acid residues forming three α-helices arranged as a barrel. Affibodies are able to withstand high temperatures (~ 90°C) and are resistant to proteolysis and to acidic and alkaline conditions (pH ranging from 2.5 to 11). A range of affibodies specific to a number of molecular targets (HER1, HER2, and TNF-α) has recently been obtained. The Z_HER2:342_ affibody (also known as ABY-002), which recognizes HER2 with K_d_ = 22 pM, is the one that has been studied most intensively [26]. The Z_HER2:342_ affibody binds to subdomain I of HER2 without competing with other compounds targeting HER2 (antibodies trastuzumab or pertuzumab), thus opening up great avenues in the theranostics of cancer.

**4.1. Modification of magnetic nanostructures with affibodies**

Affibodies are among the most efficient scaffold proteins used for targeting nanoparticles to eukaryotic cells. A comparative study addressing the efficiency of various anti-HER2-targeting molecules in delivering carboxymethyl dextran-stabilized magnetic nanoparticles (sized 25 nm) to HER2-positive cells has been conducted [66]. The affibody-modified nanoparticles are most suitable for both the magnetic detection and fluorescence imaging of cells using nanoparticles. The reason for that is the small size of Z_HER2:342_ affibody (8 kDa) compared to that of other molecules: DARPin DARP G3 (14 kDa) and trastuzumab antibody (150 kDa); so, a greater number of active molecules can be bound to the nanoparticle surface [66].

The effectiveness of affibodies is confirmed by the findings of numerous fundamental studies [100, 101]. A set of particles was produced to perform visualization and contrast-enhanced magnetic resonance imaging of EGFR- and HER2-positive cells both in vitro and in vivo. Lanthanide-doped superparamagnetic iron oxide nanoparticles sized 27 nm were obtained to perform a multiplex assay of nanoparticle–cell binding by inductively coupled plasma mass spectrometry. Anti-HER2 affibodies were conjugated to these nanoparticles using the copper-free click-chemistry method [102].

Click reactions (biorthogonal reactions) are characterized by an extremely high yield; they are regioselective and proceed under various conditions, including physiological ones. Azide-alkyne cycloaddition, with copper (I) used as a catalyst, is among the most common click reactions [103, 104, 105]. Since protein molecules typically contain neither azide nor alkyne moieties, by inserting these groups into the conjugated components and using this reaction, one obtains full control over conjugation selectivity and efficiency.

Superparamagnetic iron oxide (SPIO) nanoparticles sized 7 nm within 50-nm microemulsions formed by amphiphilic dyes (including photosensitizers), indocyanine green (ICG) and protoporphyrin IX (PpIX), were used for in vitro targeted delivery [106]. SPIO nanoparticles (sized 30 nm) modified with the anti-HER2 affibody using the click chemistry approach were employed for contrast-enhanced magnetic resonance imaging of HER2-overexpressing T6-17 tumors [107]. The number of affibody molecules bound to nanoparticles needed to be optimal, so that target recognition could be ensured and maximum contrast enhancement in MRI achieved. Thus, it was shown by determining the number of affibody molecules on the nanoparticle’s surface after the click reaction that 30-nm SPIO nanoparticles carrying 23 anti-HER2 affibody molecules on their surface (the tested range being 6–36 molecules) are the most effective ones [108].

The multifunctionality of magnetic nanostructures was also used for trimodal imaging by 24-nm 64Cu-chelated heterostructures consisting of iron oxide (Fe_3_O_4_) and gold nanoparticles. Optical, PET, and MRI imaging of EGFR-overexpressing tumors in laboratory animals was carried out using nanoparticles conjugated to the Z_EGFR:1907_ anti-EGFR affibody via the carbodiimide method [109]. Trimodal imaging of tumors by computed tomography, ultrasound imaging, and MRI was also performed. Magnetic nanoparticles sized 10 nm conjugated to Z_HER2:342_ anti-HER2 affibody and labeled with the NIR-830 near-infrared dye were used for this purpose [110, 111]. These particles, loaded with cisplatin, were subsequently used for the in vivo photothermal therapy of HER2-positive tumors [112].

Magnetic particles modified with the IGF-1R-targeted Z_IGF1R:4551_ affibody were used for both contrast-enhanced MRI and photoinduced hyperthermia of SW620 tumors upon irradiation with 808-nm light [113].

**4.2. Modification of gold nanostructures with affibodies**

Silicon-coated gold nanoparticles (sized 140 nm) modified with the Z_EGFR:1907_ anti-EGFR affibody through a heterobifunctional maleimide derivative of PEG were used to selectively label a EGFR-overexpressing cell culture and for ex vivo tumor imaging [114]. Complexes that had formed between nanoparticles and cells were detected by both fluorescence microscopy and surface-enhanced Raman scattering [114]. These nanoparticles were shown to be weakly toxic for healthy mice as confirmed by measurement of the biochemical parameters, performance of a immunohistochemical analysis, and measurement of cardiac parameters for 2 weeks after systemic delivery of nanoparticles [115]. Targeted gold nanoparticles have been designed in a number of studies for the diagnosis [110, 116] and therapy of HER2-overexpressing tumors [112, 117].

Along with their contrast-enhancement ability in Raman spectroscopy, gold nanoparticles are efficient X-ray sensitizers. Gold nanoparticles (sized 56 nm) coated with the anti-HER2 affibody were obtained using the carbodiimide method in [118]. When exposed to X-rays (at a dose of 10 Gy), these particles exhibit HER2-specific cytotoxicity; HER2-positive SK-OV-3 cells turned out to be the most sensitive cell line among the ones tested (SK-BR-3, SK-OV-3, HN-5, and MCF-7): their survival rate upon exposure to targeted nanoparticles and X-rays decreased by 63 % [118].

Au-Fe2C Janus particles sized 12 nm were synthesized to achieve the maximum efficiency in diagnosis (trimodal imaging) and therapy (photo-induced hyperthermia of the tumor). These particles were coated with the Z_HER2:342_ anti-HER2 affibody and used for in vivo trimodal tumor detection (MRI, photoacoustic imaging and computed tomography) and for in vivo 808-nm induced hyperthermia of cancer cells in HER2-overexpressing xenograft models [119].

A more elegant approach to obtaining nanoparticles with a narrow size distribution was developed based on protein nanoparticles formed by the hepatitis B virus capsid displaying affibody molecules on its surface. Gold was reduced, giving rise to gold nanoparticles sized 1–3 nm on the surface of the viral particles that had already been obtained. These EGFR-specific heterostructures sized 40 nm are effective both for cancer cell imaging via Cy5.5 labeling and for the hyperthermic effect on EGFR-overexpressing MDA-MB-468 tumor cells [120].

**4.3. Modification of the anti-Stokes nanostructures of affibodies**

Upconversion nanoparticles are efficient diagnostic tools. They allow the high-sensitivity visualization of biological objects without significant autofluorescence interference [121]. NaYF_4_:Tm^+3^,Yb^+3^ nanoparticles covalently modified with anti-EGFR affibodies have been obtained for the visualization of EGFR-expressing tumors in vivo [122]. Upconversion nanoparticles with a more complex architecture have been synthesized for photodynamic therapy of EGFR-overexpressing tumors [123]. Complex superstructures with an upconverting NaYF_4_,Yb,Er core surrounded by zinc-based organometallic framework structures were obtained. The self-assembly of such structures was performed using complementary DNA strands. When these structures are excited by external IR light, the upconverting core emits visible light, thereby exciting the organometallic frameworks that can produce reactive oxygen species and act as therapeutic agents [123].

**4.4. Affisomes**

Compounds based on affibody-conjugated liposomes are known as affisomes [124, 125]. A number of liposomes covalently modified with the ZHER2:342 anti-HER2 affibody [126] and via a polyethyleneglycol linker (Z_HER2:477_ anti-HER2 affibody [124], (Z_00477_)_2_-Cys [127], and (Z_EGFR:955_)_2_ anti-EGFR affibody [128]) have been obtained and used to treat HER2- and EGFR-positive tumors.

**4.5. Complexes of polymeric nanostructures and affibodies**

Various materials (gold, carbon, magnetite, silicon, etc.) are used for synthesizing nanoparticles. Biocompatible polymers stand out in terms of their structural and functional characteristics: e.g., poly(lactic-co-glycolic acid) (PLGA), which is already used in diagnosis and therapy. PLGA is gradually degraded to lactic and glycolic acids and is excreted from the body. Various PLGA polymers containing free carboxyl and amino groups have been synthesized, opening up avenues for particle modification with molecules that recognize tumor antigens. PLGA nanoparticles sized 140 nm and loaded with the Nile Red fluorescent dye and doxorubicin were obtained. These nanoparticles were stabilized with chitosan and conjugated to the Z_HER2:342_ anti-HER2 affibody by EDC/sulfo-NHS coupling. The PLGA–Z_HER2:342_ nanoparticles were used to label HER2-overexpressing cancer cells both in vitro and in vivo. The specificity of these nanoparticles was higher than that of the control non-targeted nanoparticles more than 60-fold. The PLGA–Z_HER2:342_ nanoparticles were used to affect the cells either alone or in combination with the DARP-LoPE-targeted bifunctional immunotoxin (42 kDa). Combination therapy using DARP-LoPE and PLGA–Z_HER2:342_ was shown to reduce the effective immunotoxin concentration 1,000-fold in vitro. This dual-targeting strategy improved the efficacy of the anti-tumor therapy of HER2-positive cells in vivo [6]. The synthesis and surface modification method was further employed to design nanoparticles loaded with a rose bengal photosensitizer agent. When irradiated at the 532-nm wavelength, these nanoparticles produce reactive oxygen species, killing HER2- overexpressing cancer cells [129].

Nanoparticles consisting of hybrid polymers are also being intensively studied. Polymeric nanoparticles formed by poly(lactide-co-glycolide)-block-poly(ethylene glycol) have been obtained, modified with the Z_HER2:342_ anti-HER2 affibody by maleimide-based chemical conjugation, and loaded with paclitaxel. These nanoparticles were used to selectively kill HER2-overexpressing cells in vitro [130].

A large number of nanoparticles (in which a polymer is the matrix for synthesizing and incorporating both soluble and insoluble compounds) have been developed. Meanwhile, the polymeric materials can per se have a diagnostic and therapeutic significance: they can possess fluorescence properties or photothermal conversion ability [131]. Thus, 30-nm nanoparticles based on polymers poly[9,9- bis(2-(2-(2-methoxyethoxy)ethoxy)ethyl)fluoroenyldivinylene]-alt-4,7-(2,1,3-benzothiadiazole) exhibiting fluorescent properties in the near-red spectral range and photosensitizing properties and poly[(4,4,9,9-tetrakis(4-(octyloxy)phenyl)-4,9-dihydro-s-indacenol-dithiophene-2,7-diyl)-alt-co-4,9- bis(thiophen-2-yl)-6,7-bis(4-(hexyloxy)phenyl)thiadiazole-quinoxaline] possessing strong near-infrared absorption and excellent photothermal conversion ability have been designed for theranostic purposes. These particles are characterized by a quantum yield of 60.4% and efficient photothermal conversion of 47.6%. The use of two types of impact (photodynamic and photothermal) was shown to have a synergistic effect in tumor therapy [132, 133]. Fluorescent hyperbranched polyelectrolyte core/shell complexes sized 30 nm were also obtained. A fluorescent polymer with the emission maximum at 565 nm, produced by polycyclotrimerization of alkynes, was used as a core; polyethylene glycol was used as a shell. These polyelectrolyte complexes were coated with an anti-HER2 affibody by carbodiimide conjugation and used as efficient fluorescent tags for the imaging of SK-BR-3 cells [134]. 

Nanobubbles, a unique class of contrast agents used for in vivo contrast-enhanced ultrasound imaging, stand out among polymeric nanomaterials [135]. Thus, 480-nm nanobubbles consisting of the phospholipid shell, filled with C_3_F_8_ gas and coated with anti-HER2 affibody using the streptavidin–biotin system have been obtained [136].

Particles of different shapes (80×320 and 55×60 nm) synthesized using the PRINT technology (particle replication in nonwetting templates) were modified with anti-EGFR affibodies with different affibody densities on the nanoparticle surface. Significant differences in the accumulation of both types of nanoparticles in the tumor depending on the affibody density were observed in vivo. The maximum ratio between the nanoparticle contents in the tumor and in blood was achieved for the particles where the amount of the ligand was maximal [137].

**4.6. Complexes of protein nanoparticles and affibodies**

In clinical practice, the biocompatibility and biodegradability of protein nanoparticles make them the leading diagnostic and therapeutic drugs. Meanwhile, the advances in genetic engineering allow us to generate fully genetically encoded fusion proteins with the desired functional characteristics without the need to use chemical conjugation techniques.

Albumin-based nanoparticles are among the most popular protein nanoparticles. They were modified with an anti-HER2 affibody using a bacterial superglue, the SpyTag (ST)/SpyCatcher (SC) protein adapter system derived from the split protein CnaB2 of Streptococcus pyogenes. SpyTag (a 13-amino acid peptide) and SpyCatcher (a 15-kDa protein) bind through a covalent peptide bond. The SpyTag/SpyCatcher system was used as a molecular mediator between the nanoparticle surface and the affibody molecule, thus ensuring that the affibody is attached regioselectively to the nanoparticle with an almost 100% efficiency. These nanoparticles were loaded with an indocyanine green photosensitizer and used for photothermally induced death of HER2-overexpressing cancer cells [138].

The SpyTag/SpyCatcher system was also successfully used to modify nanoparticles based on encapsulin [139, 140] and lumazine synthase [141]. Encapsulin (Encap) is a nanoparticle-forming protein isolated from the thermophilic bacteria Thermotoga maritima, the study of which began relatively recently. The encapsulin-SpyTag fusion protein has been obtained; this protein forms 35-nm nanoparticles with one of the elements of the adaptor system, ST [140]. Anti-HER2–anti-EGFR affibody proteins fused with the second component of the protein pair (SC) were also obtained. These fusion proteins were fluorescently tagged with two different dyes and doped with nanoparticles; specific bimodal fluorescence detection of cells characterized by different levels of HER2 and EGFR expression was then performed [140]. In a similar manner, nanoparticles based on lumazine synthase from Aquifex aeolicus (AaLS) and loaded with the gadolinium complex (Gd(III)-DOTA) were used for contrast-enhanced MRI of tumors characterized by different HER2 and EGFR expressions in mice [141].

Self-assembled protein nanoparticles (e.g., those based on hepatitis B virus capsid) are often used for both gene and protein delivery [142, 143, 144, 145, 146, 147, 148]. Viral capsid-based nanoparticles (sized 28 nm) loaded with the mCardinal far-red fluorescent protein and modified with the anti-HER2 affibody were engineered. In vivo tests showed that the particles actively accumulated in the tumor, while accumulating in the liver much less intensively compared to nanoparticles loaded with the conventional dyes (namely, Cy5.5) [142].

Human ferritin nanoparticles (sized 12 nm) consisting of 24 subunits of ferritin heavy chains fused with an anti-EGFR affibody by the genetic engineering technique have been obtained. These particles were labeled with a Cy5.5 near-red dye and used to visualize EGFR-overexpressing cells [149]. To ensure longer term in vivo circulation of ferritin nanostructures in the bloodstream, the following modifications were made: hydrophobic sequences were inserted into the structure so that a hydration shell was formed (this effect was similar to that of nanoparticle PEGylation) [150]. This approach has enhanced the accumulation of nanoparticles in the tumor twofold as confirmed by intravital imaging using the Cy5.5 dye [150].

It was found that 90-nm camptothecin-loaded mesoporous silicon nanoparticles coated with a protein corona formed by a glutathione-S-transferase/anti-HER2 affibody fusion protein bind to serum proteins to a significantly lower extent, thus minimizing the nanoparticle uptake by macrophages [151]. Such particles labeled with a DiI fluorescent dye and loaded with camptothecin, a cytotoxic quinoline alkaloid inhibiting topoisomerase I, were used for imaging and inhibiting tumor growth in vivo with 90% efficiency [151].

**4.7. Modification of tetrahedral DNA complexes with affibody molecules**

Many publications have addressed the development of systems for the targeted delivery of genetic material. For example, Z_HER2:2891_ anti-HER2 affibody molecules bound to the polyethylene glycol–polyethyleneimine copolymer were used to deliver the luciferase gene into HER2-overexpressing BT474 cells. The luminescence intensity of the transfected HER2- overexpressing cells was shown to be higher than that of the control MDA-MB-231 cells, characterized by a moderate HER2 expression of more than 300-fold [152].

DNA can carry not only genetic information, but also chemotherapeutic drugs. In particular, DNA tetrahedra (3D structures produced from 20 bp DNA double helices using the DNA origami method) act as scaffolds. DNA tetrahedra chemically modified with the anti-HER2 affibody via maleimide conjugation and loaded with doxorubicin (53 doxorubicin molecules per complex) [153] inhibited cell growth significantly stronger compared to doxorubicin, while being much less toxic to cells with a normal HER2 expression level. Similar cisplatin-loaded nanoparticles (68 cisplatin molecules per nanoparticle) were used to selectively kill HER2-positive cells with an almost 100% efficiency level [154].

A fusion protein consisting of the Z_HER2:342_ affibody and RALA peptide, an efficient nonviral agent for nucleic acid delivery into cells, was also obtained. The affibody and the peptide were connected by a flexible protease-resistant glycine–serine linker (G_4_S)_3_. The resulting fusion peptide is associated with FUdR_15_, a sequence of 15 residues of 5-fluorodeoxyuridine that is metabolized into a 5-fluorouracil chemotherapeutic agent [155]. The resulting system has a targeted impact on HER2-overexpressing N87 cells and leads to their apoptosis [155]. Subsequently, the targeted delivery mechanisms elaborated in the reviewed studies [154, 155] were combined into the DNA tetrahedron-based system for the delivery of FUdR to the cells of a tumor induced by the injection of BT474 cells; this system slowed down tumor progression approximately 2.5-fold [156].

**4.8. Modification of quantum dots with affibodies**

Quantum dots are fluorescent semiconductor nanocrystals (with a core sized 1–12 nm) synthesized from group II and VI elements (e.g., ZnS, CdSe or CdTe) or, less frequently, group III and V (InP) or group IV and VI (PbS, PbSe, or PbTe) elements. They differ from the conventional fluorophores such as organic dyes and fluorescent proteins in terms of their broad absorption band, significant Stokes shift, narrow emission spectrum, and high quantum yield (up to 80%), as well as high photostability [157, 158]. The significant dependence of the emission wavelength on the particle size makes it possible to perform multicolor labeling and simultaneous identification of different biological objects. However, the toxicity of QDs significantly limits the scope of their in vivo application for therapeutic purposes. The use of QDs for sentinel lymph node mapping is much more promising, since in this case the drug is injected locally and the metastatic lymph node is subsequently resected.

In particular, quantum dots QD655 modified with the Z_HER2:477_ anti-HER2 affibody through the streptavidin–biotin system have been used for diagnostic purposes. These quantum dots have been applied for the immunohistochemical staining of tumor cross-sections to successfully identify the HER2 status of the tumor, as well as the presence and localization of HER2 homodimers, by confocal and electron microscopy [159, 160].

Quantum dots QD800 sized 5 nm (core/shell/shell = InAs/InP/ZnSe) conjugated to the Z_HER2:342_ anti-HER2 affibody through a heterobifunctional PEG derivative carrying a terminal amino group were used for in vivo imaging. The affibody was modified with cysteine at its N-terminus, and the chemical conjugation reaction was performed using 4-maleimidobutyric acid N-succinimidyl ester. Anti-HER2 quantum dots were employed for selective real-time imaging of SK-OV-3 tumors in immunodeficient mice using an intravital imaging system [161]. The accumulation of targeted quantum dots in the tumor was shown to be approximately threefold higher than that of non-targeted ones [161].

Z_EGFR:1907_ anti-EGFR affibody was adsorbed onto the surface of 8 nm silver sulfide (Ag_2_S) quantum dots, and the modified particles were used for photoacoustic imaging of EGFR-overexpressing tumors [162]. The same quantum dots coated with IGF-1R-recognizing affibody, ZIGF1R, were used in vivo for bimodal photoacoustic imaging and near-infrared imaging of tumors in immunodeficient animals [163].

Carbon dots possessing a broad range of unique optical characteristics have found a wide application. Thus, not only do 20 nm gadolinium-encapsulated Gd@C carbon dots possess bright fluorescence, but they also exhibit MRI contrast properties [164]. These dots were coated with Z_EGFR:1907_ anti-EGFR affibody and used for both in vitro and in vivo targeted delivery. It was shown in vitro that the MRI signal for HCC827 cells (EGFR+) is significantly higher than that for NCI-H520 cells (EGFR-). These structures are also efficient for in vivo targeted tumor imaging 1 h post-injection (MRI signals for HCC827 and NCI-H520 tumors differed by a factor of 1.5). Furthermore, Gd@C quantum dots with Z_EGFR:1907_ are efficiently excreted by the kidneys, unlike Gd@C dots [164].

## 5. TARGETED ANTIBODIES BASED ON ADAPT PROTEINS

The high affinity constants of proteins based on albumin-binding domains (ABDs) ADAPT have made it possible to design an ultrasensitive method for detecting HER2 in the samples containing 10% of serum. Thus, QD625 quantum dots have been obtained and modified by HER2-targeting ADAPT6 via self-assembly. The threshold of HER2 detection using these quantum dots was 40 × 10^−2^ M (≈8 ng/mL) [165].

## 6. CONCLUSIONS

Scaffold proteins can be called next-generation proteins [166, 167, 168, 169]. An appreciably large number of medications based on these proteins are currently undergoing clinical trials [170, 171, 172, 173, 174, 175], and some of them are already used in theranostics (e.g., ecallantide, a protein based on the Kunitz domain).

Despite such advantages as small size, stable structure, and the simplicity of large-scale biotechnological production, these proteins also have shortcomings when used in combination with functional nanostructures, which are related to regioselective binding to the surface of nanostructures, while the recognition properties are retained. The problems of this kind are solved using various molecular mediators between the nanoparticle surface and protein molecules (e.g., SpyTag–SpyCatcher, barnase/barstar, and streptavidin/biotin), as well as genetic engineering techniques (e.g., incorporation of DARPins into the viral envelope).

Our advances in chemical modification and genetic engineering allow one to produce nanoparticles that are maximally effective only in vitro. When targeted nanoparticles are injected systemically into the bloodstream, their accumulation in the tumor is often no more than 2.5 times greater than that in the case of non-targeted nanoparticles; the total accumulation in the tumor is no greater than 0.7% of the injected dose.

Along with the development of targeted agents for the therapy and diagnosis of cancer (as well as cancer theranostics), designing novel methods for nanoparticle administration and delivery is an equally important task in nanobiomedicine. This has received much less attention thus far. In particular, methods for prolonging nanoparticle circulation in the bloodstream are being developed: the mononuclear phagocyte system is suppressed temporarily without any serious side effects.

Since solid tumors are dense heterogeneous structures, the in vivo impact of targeted therapeutic agents on cancer cells is meaningful only for the uppermost tumor layers, while deep-lying cells remain viable, thereby neutralizing the effect of the targeted action. Angiogenesis needs to be inhibited (through their impact on endothelial markers), thus disrupting the blood supply to deep-lying cancer cells.

## Abbreviations

ADP: adenosine diphosphate
LSPR: localized surface plasmon resonance
MAPK: mitogen-activated protein kinase
MRI: magnetic resonance imaging
MNPs: magnetic nanoparticles
PMAO: poly(maleic anhydride/1-octadecene)
PEG: polyethylene glycol
PET: positron emission tomography
RNAse: ribonuclease
ADAPT: albumin-binding domain-based scaffold protein
Bs C Mms6: fusion protein of barstar with C Mms6
DARP: designed ankyrin repeat protein
DARP 9_29 Bn: fusion protein of DARPin 9_29 with barnase
DARP-LoPE: fusion protein of DARPin 9_29 with LoPE
DOTA: dodecanetetraacetic acid
EDC: 1-ethyl-3-(3-dimethylaminopropyl) carbodiimide
eEF2: eukaryotic elongation factor 2
EGF-1R: insulin-like growth factor 1 receptor
EpCAM: epithelial cell adhesion molecule
EPR: enhanced permeability and retention effect
HER2: human epidermal growth factor 2 receptor
IgE: immunoglobulin E
IgG: immunoglobulin G
NHS: hydroxysuccinimide
PE, ETA: pseudomonas exotoxin A of Pseudomonas aeruginosa
PRINT: particle replication in nonwetting templates
ScFv: a single-chain fragment of the light and heavy chains of immunoglobulin
SBP: silica binding peptide
SPIO: superparamagnetic nanoparticles
TNF-α: tumor necrosis factor
VEGF-A: vascular endothelial growth factor.
